# ACE polymorphism and diabetic nephropathy

**DOI:** 10.4103/0971-4065.65313

**Published:** 2010-04

**Authors:** V. Wiwanitkit

**Affiliations:** Wiwanitkit House, Bangkhae, Bangkok, Thailand - 101 60

Sir,

I read with great interest the recent report by Naresh *et al*.[1] Naresh *et al*. finally suggested an association of DD polymorphism and type II diabetes with nephropathy."[1] Indeed, angiotensin converting enzyme (ACE) polymorphism is a classical, widely studied genetic polymorphism. It is confirmed that ACE polymorphism is correlated to the progression on renal disease.[2] However, there are some interesting notes for the present work. First, it should be noted that the genetic polymorphism is a genetic topic to be talked about on a population scale. A common limitation of genetic polymorphism is the limitation in the number of subjects. Second, race might have a significant effect on the pattern of ACE polymorphism.[3] This data should be clarified and assessed. Finally, it should also be cited that the recent data on metaanalysis for the correlation between ACE polymorphism and diabetic retinopathy, another common diabetic complication, showed null correlation.[4]
